# Molecular, Histological, and Functional Changes in Acta1-MCM;FLExDUX4/+ Mice

**DOI:** 10.3390/ijms252111377

**Published:** 2024-10-23

**Authors:** Solene Sohn, Sophie Reid, Maximilien Bowen, Emilio Corbex, Laura Le Gall, Eva Sidlauskaite, Christophe Hourde, Baptiste Morel, Virginie Mariot, Julie Dumonceaux

**Affiliations:** 1 NIHR Biomedical Research Centre, University College London, Great Ormond Street Institute of Child Health and Great Ormond Street Hospital NHS Trust, London WC1N 1EH, UK; s.sohn@ucl.ac.uk (S.S.); sophie.reid@ucl.ac.uk (S.R.);; 2 Laboratoire Interuniversitaire de Biologie de la Motricité LIBM, EA 7424, Savoie Mont Blanc University, F-7300 Chambéry, France

**Keywords:** FSHD, DUX4, mouse model, muscle, myopathy, biomarker, force test, strength

## Abstract

DUX4 is the major gene responsible for facioscapulohumeral dystrophy (FSHD). Several mouse models expressing DUX4 have been developed, the most commonly used by academic laboratories being ACTA1-MCM/FLExDUX4. In this study, molecular and histological modifications in the tibialis anterior and quadriceps muscles were investigated in this model at different time points. We investigated several changes that could be used as markers of therapeutic efficacy. Our results confirm the progressive muscular dystrophy previously described but also highlight biases associated with tamoxifen injections and the complexity of choosing the genes used to calculate a DUX4-pathway gene composite score. We also developed a comprehensive force test that better reflects the movements made in everyday life. This functional force–velocity–endurance model, which describes the force production capacities at all velocity and fatigue levels, was applied on 12–13-week-old animals without tamoxifen. Our data highlight that previously unsuspected muscle properties are also affected by the expression of DUX4, leading to a weaker muscle with a lower initial muscle force but with preserved power and endurance capacity. Importantly, this force–velocity–endurance approach can be used in humans for clinical evaluations.

## 1. Introduction

Facioscapulohumeral dystrophy (FSHD) is a rare genetic disease with a prevalence of 4.5 out of 100,000 in Europe [1]. The disease is characterised by weakness and atrophy of specific muscle groups [2]. The likely cause of the disease is the aberrant expression of the double homeobox DUX4 transcription factor in skeletal muscles [3,4], whose ORF is in a 3.3 kb tandemly repeated sequence (D4Z4) located in the 4q subtelomeric region [5]. DUX4 is usually expressed during embryonic development to regulate zygotic genome activation in placental mammals [6], but it is epigenetically silenced in most somatic tissues after birth [6]. FSHD patients show a loss of repressive epigenetic marks at the D4Z4 repeats [7,8,9], correlated with disease severity [10,11] and leading to the aberrant expression of DUX4 in muscles. This hypomethylation can be the consequence of either a reduction in the D4Z4 copy number in the subtelomeric part of chromosome 4 [12,13] and/or mutations in epigenetic modifier genes, including SMCHD1, DNMT3b, and LRIF1 [14,15,16]. DUX4 has already been detected in foetal FSHD muscle biopsies [4,17], slowly damaging the muscle fibres, ultimately leading to FSHD pathology. In muscles, DUX4 is a toxic protein that affects different pathways, triggering apoptosis and necroptosis [18,19,20,21], ultimately leading to muscle weakness and atrophy [22]. All these data have made DUX4 a prime target for therapeutic approaches, and over the last 10 years, several laboratories, including ours, have been looking for different ways to inhibit DUX4 expression [23,24]. The three transgenic animal models that are currently used in most FSHD laboratories worldwide are 1—the Acta1-MCM;FLExDUX4/+ double-transgenic mouse line, which carries DUX4 exons 1 to 3 in an antisense orientation to the Rosa26 promoter, and the mER-Cre-mER recombinase gene under the control of the muscle-specific ACTA1 promoter [25,26]; 2—the iDUX4pA-HSA mouse carrying a 2.7 kb DUX4 gene fragment containing the DUX4 ORF and the 3′UTR sequence including DUX4 poly(A) under the control of a doxycycline-inducible promoter [27]; and 3—the TIC-DUX4 mouse, which is also a double-transgenic animal carrying the DUX4 gene and pLAM sequence but with an additional poly(A) from the bovine growth hormone and the HSA-mER-Cre-mER transgene [28]. These three models show high levels of DUX4 expression after the addition of tamoxifen [25,26,28] or doxycycline [27] but can also develop a long-term chronic disease [29,30]. Both Acta1-MCM;FLExDUX4/+ and TIC-DUX4 mice have been used to develop therapeutic approaches for DUX4 [28,31,32,33,34], as well as the FLExDUX4 mouse model [35,36], which only carries the DUX4 gene cloned in an antisense orientation [25,37].

The identification of DUX4 as the major gene of FSHD has led biopharma and academic laboratories to the development of therapeutic approaches targeting DUX4. The development of these approaches inevitably involves an in vivo evaluation. The articles published by Nunes et al. [29] and Jones et al. [26] have shown that, even in the absence of tamoxifen, the Acta1-MCM;FLExDUX4/+ mouse presents chronic mosaic DUX4 expression, developing a DUX4-induced pathology, which reflects the course of the disease in FSHD patients. Mice six months old and over show clear phenotypic and molecular differences compared to age-matched ACTA1-MCM/+ mice, but it is less clear in younger animals. Having a robust animal model in which histological and molecular changes are visible at young ages and have functional effects would be ideal. In this study, we investigated histological and molecular changes in the FLExDUX4/+ and Acta1-MCM;FLExDUX4/+ mouse models at a young age, as these two models were used to evaluate therapeutic approaches. We developed innovative in vivo force tests based on a functional force–velocity–endurance model for the Acta1-MCM;FLExDUX4/+ mouse that can be used in mice as young as 12 weeks without tamoxifen.

## 2. Results

### 2.1. Acta1-MCM;FLExDUX4/+ Animals Present a Slow Dystrophic Process, with the Quadriceps Muscle Being More Affected than the Tibialis Anterior

**Figure 1:**
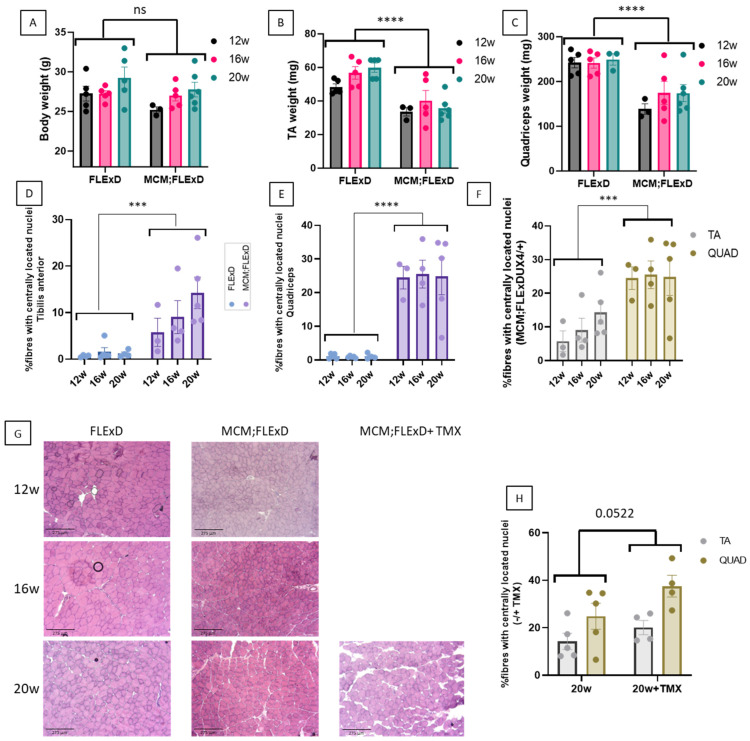
Acta1-MCM;FLExDUX4/+ animals present a slow dystrophic process. The total body weight (**A**), TA weight (**B**), QUAD weight (**C**), and the percentage of centrally located nuclei in the TA (**D**,**F**) and the QUAD (**E**,**F**) were measured at 12, 16, and 20 weeks in males. Transverse frozen sections were performed on the TA at different ages, and sections were stained with hematoxylin and eosin (**G**). In some animals, TMX (2.5 mg/kg weekly for 4 weeks) was injected (**G**,**H**). Error bars indicate the mean of SEM of 3–5 biological replicates. *p*-values were calculated using GraphPad Prism9, 2-way ANOVA. A post hoc test was not performed as no interaction between age and genotype was found. *** *p* < 0.001; **** *p* < 0.0001.

To determine which histological and molecular changes are the most visible at young ages, several parameters were compared in the Acta1-MCM;FLExDUX4/+ and FLExDUX4/+ animals at the age of 12, 16, and 20 weeks in males. Results for females are presented in Supplementary Figures. In some Acta1-MCM;FLExDUX4/+ animals, tamoxifen was injected at 16 weeks and the mice were sacrificed 4 weeks later. The body weights of the Acta1-MCM;FLExDUX4/+ and FLExDUX4/+ mice did not differ significantly between males and females (Figure 1A and Supplementary Figure S1A). We mainly focused on the tibialis anterior (TA) and the quadriceps (QUAD) for the histological analyses and observed that the weights of both the TAs and QUADs were statistically different when the FLExDUX4/+ and Acta1-MCM;FLExDUX4/+ mice were compared, but no difference was observed with age (Figure 1B,C and Supplementary Figure S1B,C). In terms of histological changes, we examined several parameters. The number of fibres with centrally located nuclei, a phenomenon that is often caused by muscle wasting and muscle degeneration/regeneration, was significantly increased in both males and females in the TA and QUAD of Acta1-MCM;FLExDUX4/+ animals (Figure 1D,E,H and Supplementary Figure S1D,E,H). The percentage of fibres with centrally located nuclei in Acta1-MCM;FLExDUX4/+ animals was greater in the QUAD than in the TA (Figure 1F and Supplementary Figure S1F). TMX injection resulted in a higher number of centrally located nuclei (Figure 1H and Supplementary Figure S1H).

**Figure 2:**
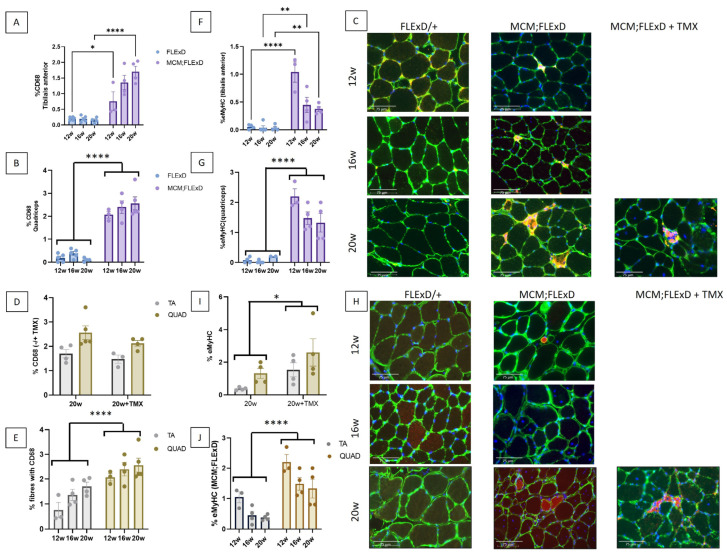
Acta1-MCM;FLExDUX4/+ present inflammation and regenerative fibres. The percentage of fibres positive for CD68 staining (**A**–**E**) and the percentage of eMyHC-positive fibres (**F**–**J**) were measured in the TA (**A**,**F**) and the QUAD (**B**,**G**) at 12, 16, and 20 weeks in FLExDUX4/+ and ACTA1-MCM/+;FLExDUX4/+ (MCM;FLExD) males. In some animals, TMX (2.5 mg/kg weekly for 4 weeks) was injected (**C**,**D**,**H**,**I**). The number of fibres positive for CD68 staining or eMyHC was compared in the TA and QUAD muscles (**E**,**J**, respectively). Scale bar: 75 μm (**C**,**H**). Error bars indicate the mean of SEM of 3–5 biological replicates. *p* values were calculated using GraphPad Prism9 and 2-way ANOVA followed by Fisher’s LSD post hoc test. The statistical significance between 12-, 16-, and 20-week-old animals is presented only when there is an interaction between genotype and age (**A**,**F**). * *p* < 0.05; ** *p* < 0.01; **** *p* < 0.0001; TMX: tamoxifen.

TA and QUAD sections were stained with the pan-macrophage marker CD68 to determine the extent of macrophage infiltration of the skeletal muscle. The TA and QUAD of FLExDUX4/+ animals showed minimal macrophage infiltration (less than 0.3%), whereas in the Acta1-MCM;FLExDUX4/+ animals, up to 3.5% CD68-positive fibres were observed (Figure 2A–C and Supplementary Figure S2A–C). This number was further increased in the presence of TMX in females (Supplementary Figure S2D). The QUAD presented greater macrophage infiltrations than the TA (Figure 2E and Supplementary Figure S2E).

The number of fibres positive for eMyHC, a marker for actively regenerating myofibers, was also investigated. Few eMyHC-positive fibres were found in the FLExDUX4/+ animals (Figure 2F–H and Supplementary Figure S2F–H). TMX worsened the eMyHC phenotype (Figure 2I and Supplementary Figure S2I). Again, the QUAD muscle presented an increased number of eMyHC as compared to the TA (Figure 2J and Supplementary Figure S2J), thus confirming that the QUAD was more affected than the TA.

**Figure 3:**
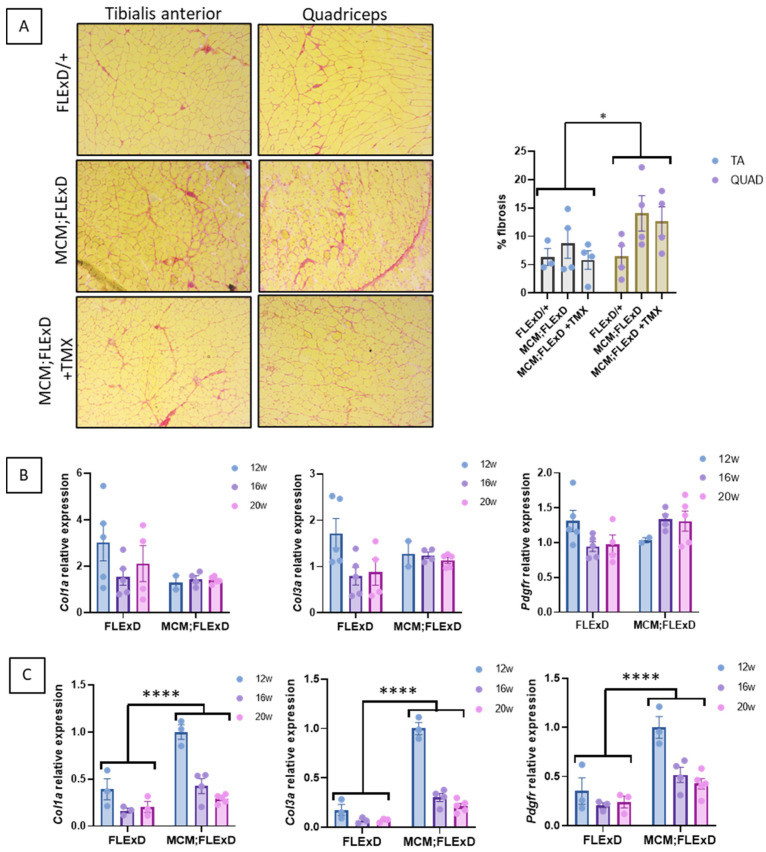
Acta1-MCM;FLExDUX4/+ mice present fibrotic infiltration. Acta1-MCM;FLExDUX4/+ or FLExDUX4/+ males were treated or not with tamoxifen at the age of 16 weeks and killed at the age of 20 weeks. Picrosirius red was used to stain collagen on the TA (upper panel) and QUAD (lower panel) sections (**A**) in animals killed at the age of 20 weeks. The expression of several genes involved in fibrosis was investigated in the TA (**B**) and QUAD (**C**) in males aged 12, 16, or 20 weeks. Error bars indicate the mean of SEM of 3–5 biological replicates. *p* values were calculated using GraphPad Prism9 and 2-way ANOVA followed by Fisher’s LSD post hoc test. * *p* < 0.05; **** *p* < 0.0001; TMX: tamoxifen.

Fibrosis infiltration was also investigated, and the presence of collagen bundles revealed it. A more pronounced infiltration was observed in the QUAD as compared to the TA of males only (Figure 3A and Supplementary Figure S3A). Compared to the FLExDUX4/+ mice, the Acta1-MCM;FLExDUX4/+ mice presented higher expression levels of the genes involved in fibrosis (*Col1a*, *Col3a* and *Pdgfra*) in their QUAD but not in their TA (Figure 3B,C and Supplementary Figure S3B,C). This is consistent with the more pronounced histological deterioration observed in the QUAD. When the expression of these genes was compared in the Acta1-MCM;FLExDUX4/+ mice with age, the effects of age, muscle, sex, age x sex, and muscle x sex were estimated (Supplementary Figure S3A). The injection of TMX at the age of 16 weeks did not change the expression levels of these three genes at 20 weeks (Supplementary Figure S4B).

In conclusion, both the TA and QUAD muscles present a slow degenerative process without tamoxifen, thus confirming the results published by the Jones group [25,26,29]. The QUAD muscle appears more affected than the TA, corresponding with the 5% and 10% transgene recombination observed in the TA and QUAD muscles, respectively, in the absence of tamoxifen [26].

### 2.2. DUX4-FL2 mRNA Is Overexpressed in Muscles of ACTA1-MCM;FLExDUX4/+ Mice

**Figure 4:**
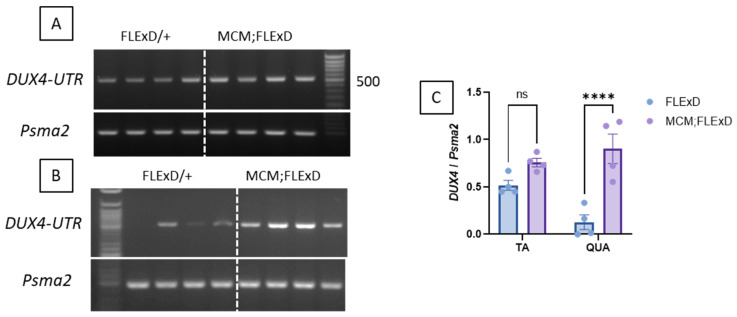
*DUX4* is overexpressed in Acta1-MCM;FLExDUX4/+ mice. Representative RT-PCR analysis of DUX4 expression from either TA (**A**) or the QUAD (**B**) muscles from FLExDUX4/+ or Acta1-MCM;FLExDUX4/+ mice using the DUX4-UTR set allows the distinct amplification of the *FL1-* and *FL2-DUX4* isoforms (product size: 368 bp and 504 bp for FL1 and FL2 respectively). *Psma2* was used as a normaliser. PCR products were run on a 2% agarose gel. Band intensities were analysed using ImageJ software 1.51j8 (**C**). Error bars indicate the mean of the SEM of four different muscles. *p* values were calculated using Graphpad Prism9 and 2-way ANOVA followed by Fisher’s LSD post-test. **** *p* < 0.0001.

To determine if the TA and QUAD express different levels of DUX4mRNA or different isoforms, we performed a PCR using primers to amplify the two DUX4-FL isoforms, namely DUX4-FL1, in which intron 1 is spliced (leading to a 368 bp amplicon), and DUX4-FL2, which includes the non-spliced intron 1 (504 bp amplicon). It is worth noting that the DUX4 genomic sequence was mutated to inhibit the production of the DUX4-s isoform in the FLExDUX4/+ animals [25]. DUX4-FL1 was not detected in any samples. DUX4-FL2 was observed in all the TA samples (Figure 4A) and most of the QUAD samples (Figure 4B). In the QUAD, DUX4-FL2 was expressed at very low levels in FLExDUX4/+ but dramatically increased in Acta1-MCM;FLExDUX4/+ mice (*p* = 0.009; Figure 4C).

### 2.3. Expression of the Genes Downstream of DUX4 Is Impacted by Several Parameters

**Figure 5:**
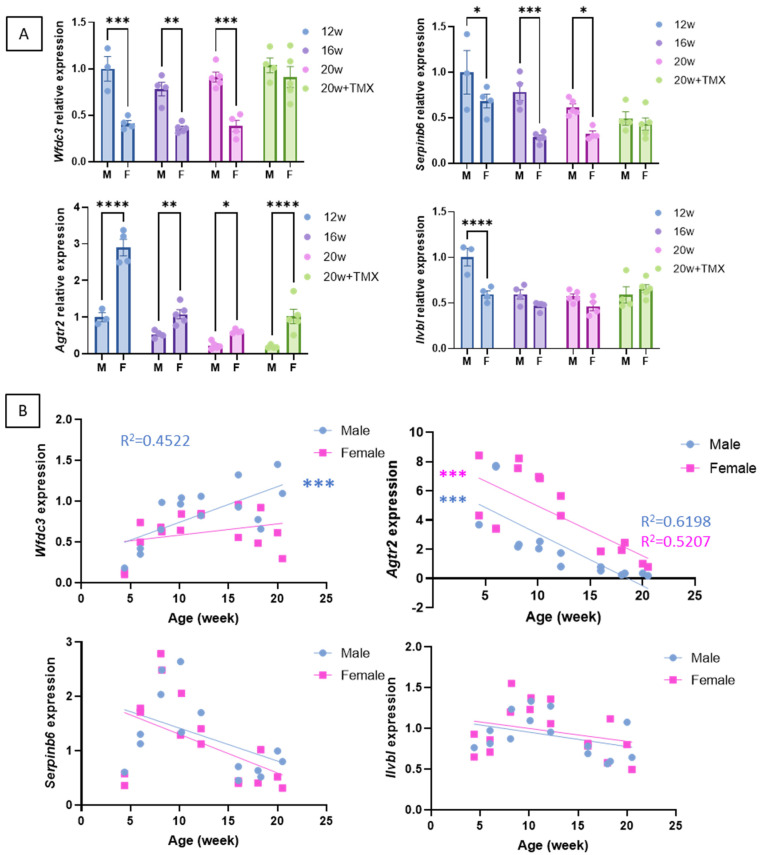
Expression of the genes downstream of DUX4 in the QUAD muscle of Acta1-MCM;FLExDUX4/+ males and females The expression levels *Wfdc3*, *Agtr2*, *Serpinb6*, and *Ilvbl* were compared at 12, 16, or 20 weeks and in the absence or presence of tamoxifen (**A**). Error bars indicate the mean of the SEM of 3–5 biological replicates. *p*-values were calculated using GraphPad Prism9 and 2-way ANOVA followed by Fisher’s LSD post hoc test. In (**B**), the Wfdc3, Agtr2, Serpinb6, and Ilvbl levels were followed from 4 to 20 weeks of age in Acta1-MCM;FLExDUX4/+ males or females. *p* values were calculated using GraphPad Prism9 after a simple linear regression analysis. A low *p*-value (<0.05) means that the slope will likely not equal zero. R^2^ is indicated on each graph *p* < 0.05. TMX: tamoxifen; M: male; F: female. * *p* < 0.05; ** *p* < 0.01; *** *p* < 0.001; **** *p* < 0.0001.

The expression of the four classically used DUX4 response genes, *Wfdc3*, *Agtr2*, *Serpinb6*, and *Ilvbl*, was analysed in males and females. All these genes were expressed at very low levels in the FLExDUX4/+ mice (Supplementary Figure S5), thus confirming that *DUX4* mRNA is not translated into a functional DUX4 protein without Cre. In the presence of Cre, all these genes were highly expressed (Supplementary Figure S5). To determine if other factors besides DUX4 can influence the expression of the genes downstream of DUX4, we performed a more comprehensive analysis. In the QUAD muscle, we observed that males and females express these genes differently: while females expressed less *Wfdc3* and *Serpinb6* than males, they expressed more *Agtr2* than males (Figure 5A). In the TA, the male–female difference is less clear, which could be linked to the lesser severity observed in the TA (Supplementary Figure S6A). Moreover, the injection of TMX did not lead to a higher expression of *Agtr2*, *Serpinb6*, and *Ilvbl* (Figure 5A) but smoothed out the sex-based difference observed for *Wfdc3* and *Serpinb6*. We also observed a correlation between the levels of *Wfdc3* and *Agtr2* and the age of the mice, with older mice expressing more *Wfdc3* (range 4–20 weeks) and less *Agtr2* (Figure 5B and Supplementary Figure S6B).

These results highlight the complexity of choosing the genes that are used to calculate a DUX4-pathway gene composite score, such as the need to consider the gene’s sensitivities to different parameters. They emphasise biases associated with tamoxifen injections (see discussion).

### 2.4. The Expression of DUX4 Led to a Decreased Absolute In Situ Force Capacity for the Tibialis Anterior Muscle Partly Due to Loss of Muscle Mass

**Figure 6:**
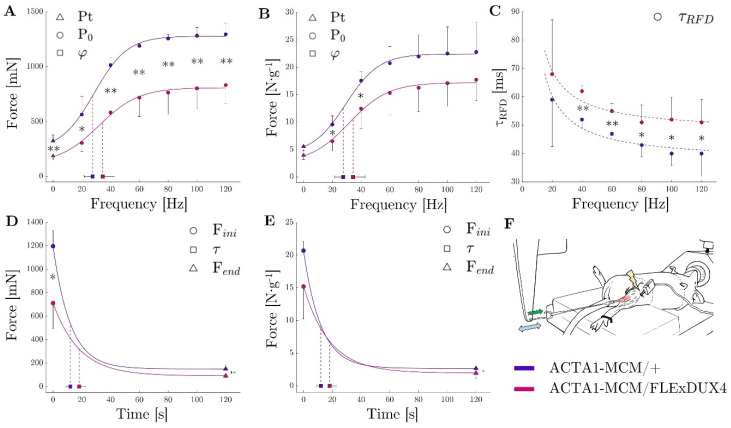
TA muscle force evaluation in ACTA1-MCM/+ (purple) and ACTA1-MCM/FLExDUX4 mice (pink). Peak Twitch (Pt; frequency = 0 Hz) and maximum tetanic force (P_0_) for stimulation from 20 to 120 Hz expressed in absolute force (mN; **A**) and relative to muscle mass (in N ^−1^; **B**); τ RFD (time to reach 63% of the tetanic force) for stimulation from 20 to 120 Hz expressed in seconds (**C**); force decrease during the 2 min tetanic stimulation at 60 Hz represented by the initial force (F_ini_), the end-test force (F_end_), and the time to reach 63% of the decrease (Tau, τ) expressed in absolute force (mN; **D**) and relative to muscle mass (**E**); experimental apparatus (**F**) (Green arrow represent the force apply by the muscle on the lever arm; Blue double-arrow represent the back and forth movement of the lever arm); *p* values were calculated using Graphpad Prism9: 2-way ANOVA followed by Fisher’s LSD post hoc test were performed. * *p* < 0.05; ** *p* < 0.01.

One of the critical readouts is the functional assessment, as measurements of muscle function are crucial to the assessment of potential treatments for muscular diseases. When compared to the Acta1-MCM/+ mice, the Acta1-MCM;FLExDUX4/+ mice presented a much lower tetanic force capacity whatever the stimulation frequency (all P0 significantly different, average difference: −43% (Figure 6A)). This decrease in force was only partly due to a reduction in the mass of the TAs (Acta1-MCM;FLExDUX4/+: 47 ± 3 mg vs. Acta1-MCM/+: 59 ± 8 mg; *p* = 0.043). Indeed, P0s, which is the maximum specific tetanic force (normalised to the weight of the muscles), was not different in the two groups for simulation frequencies equal to or above 60 Hz (Figure 6B). During a single stimulation or at lower stimulation frequencies (Pts or P0s at 20 and 40 Hz), the specific forces of the TA of Acta1-MCM;FLExDUX4/+ mice were ≈ −30% (E.C. ± 2%) lower than those observed in the Acta1-MCM/+ mice (−29%, *p* = 0.015; −32%, *p* = 0.045; −29%, *p* = 0.045, respectively) (Figure 6B). This is in accordance with parameters obtained from the fitting of the force–frequency relationship (median r^2^ = 0.956). Indeed, the largest ϕ (the frequency at which 63% of the maximum force is reached) was observed in Acta1-MCM;FLExDUX4/+ mice (+24%, *p* = 0.208, Figure 6B), although the statistical power was not sufficient to highlight a significant difference, if any. The normalised rate of force development was evaluated by the time required to reach 63% of the maximum tetanic force for a given stimulation frequency: τ RFD. The latter was lower in the Acta1-MCM;FLExDUX4/+ animals by 18 ± 4% on average when considering all stimulation frequencies (Figure 6C).

### 2.5. ACTA1-MCM;FLExDUX4/+ Mice Showed Greater Fatigue, Albeit at a Slower Rate of Onset

Considering the sustained 60 Hz stimulation, the Acta1-MCM;FLExDUX4/+ mice took longer to reach a 30% force loss (Acta1-MCM;FLExDUX4/+: 8 ± 2 s vs. Acta1-MCM/+: 5 ± 2 s; *p* = 0.056). This is consistent with the τ (curvature constant of the exponential modelling of the force decay) showing a tendency to be higher in the Acta1-MCM;FLExDUX4/+ animals (+51%, *p* = 0.064, Figure 6D). Altogether, this shows a slower rate of fatigue in Acta1-MCM;FLExDUX4/+ mice. Conversely, at the end of the 2 min contraction protocol, both the absolute (in mN) and specific (in N.g^−1^) tetanic forces were lower in the Acta1-MCM;FLExDUX4/+ mice compared to the Acta1-MCM/+ mice (−46%, *p* = 0.001 and −34%, *p* = 0.028 respectively, Figure 6E). The asymptote of the force–time relationship was also lower in the Acta1-MCM;FLExDUX4/+ group both for absolute and specific force (−39%, *p* = 0.022 and −26%, *p*= 0.122, respectively). Taken together, this shows a force fatigability that seems independent of loss of muscle mass.

### 2.6. A Comprehensive Functional Force–Velocity–Endurance Model Reveals Muscle Properties Affected by the Expression of DUX4, Leading to a Muscle with Less Force Capacity but Which Seems to Have Special Features That Enable It to Maintain Its Power Capacity Both in a Fresh and Fatigued State

We developed a force–velocity–endurance (FoVE) model to in situ compare the muscle capacities of the ACTA1-MCM/+ and ACTA1-MCM;FLExDUX4/+ transgenic mice. The FoVE model fitting to experimental data was excellent (median r^2^ = 0.970). The seven FoVE parameters are F_0i_, F_0c_, V_0i_, V_0c_, C_i_, C_c_, and τ. F_0_ represents the maximum force capacity of the muscle in the isometric condition (null velocity). V_0_ represents the highest velocity at which the muscle can transmit force. C corresponds to the curvature of the F(v) relationship. The greater the C, the more curved the F(v) relationship and the greater the force loss for a given velocity. The initial (_i_) and critical (_c_) capacities represent the fresh maximum and extreme fatigue capacities, respectively. τ is the characteristic decay time. It should represent the rate at which the fatigue occurs (at τ seconds, ≈63% of the final decay is reached).

**Figure 7:**
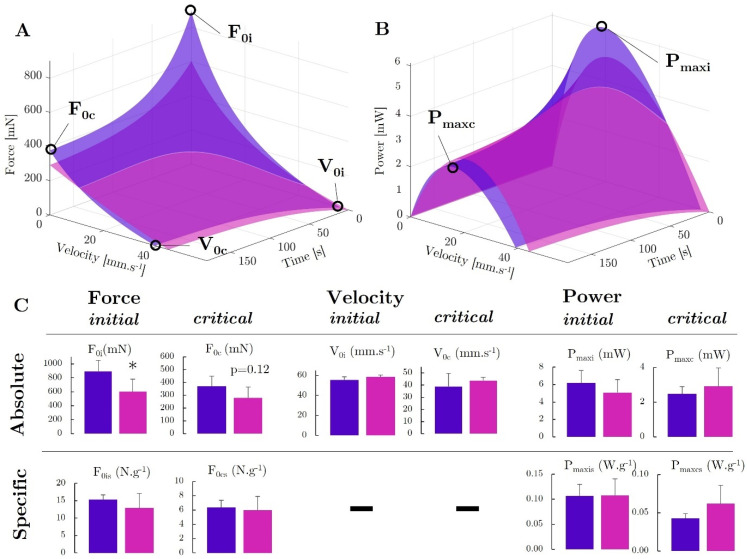
*Force–velocity–endurance (FoVE)* evaluation in ACTA1-MCM/+ (purple) and Acta1-MCM;FLExDUX4/+ mice (pink). The 3D graphical representation is presented for the relationship of force as a function of the contraction velocity and time (**A**) and power as a function of the contraction velocity and time (**B**). The main indices are represented graphically. F_0_: muscle maximal force in isometric condition (i.e., static, null velocity); V_0_: muscle maximum contraction velocity; P_max_: muscle maximal power production capacity. Indices i and c, represent, respectively, the initial condition in a fresh state, ending in extreme fatigue at the end of the test. Statistical comparison of FoVE indices expressed in absolute and specific terms, i.e., normalised to muscle mass (**C**). *p* values were calculated using GraphPad Prism9: 2-way ANOVA followed by Fisher’s LSD post hoc test were performed. * *p* < 0.05.

First, in line with the previously described force evaluation, FoVE evaluation showed that (i) the maximal initial force capacity (F_0i_) was lower in the Acta1-MCM;FLExDUX4/+ animals than in the Acta1-MCM/+ ones (−33%, *p* = 0.049) but not when normalised to muscle mass (*p* = 0.195, Figure 7A,C); (ii) τ was higher in the Acta1-MCM;FLExDUX4/+ animals (+34%, *p* = 0.008, Supplementary Figure S7); (iii) critical force (i.e., force at the end of the fatiguing test; F_0c_) tended to be lower for the Acta1-MCM;FLExDUX4/+ (−25%, *p* = 0.122) but not when normalised to muscle mass (Figure 7A,C). In addition, the FoVE evaluation can be used to characterise other functional capabilities not assessed by traditional force tests. In particular, it is possible to characterise the maximum muscular velocity and power generation capacities. None of these differed between the two groups. The TA of the Acta1-MCM;FLExDUX4/+ mice showed no significant difference compared to the Acta1-MCM/+ mice for the maximum initial and critical powers (P_maxi_: −18%, *p* = 0.204; P_maxis_ +1%, *p* = 0.485; P_maxc_ +17%, *p* = 0.247; P_maxcs_ +45%, *p* = 0.125; Figure 7B,C) as well as velocities (V_0i_: +5.7%, *p* = 0.094; V_0c_ +12.5%, *p* = 0.244; Figure 7). Furthermore, the curvature (C) of the force–velocity relation can be estimated. Without fatigue, the initial curvature C_i_ was not significantly different between the two groups (C_i_: −27.3%, *p* = 0.188).

However, at the end of the protocol, when the fatigue was the highest, a tendency towards a flatter force–velocity relationship (i.e., lower C_c_) was observed in the Acta1-MCM;FLExDUX4/+ animals (C_c_: −85.6%, *p* = 0.111; Supplementary Figure S7). Despite a lower force capacity, the less pronounced curvature of the Acta1-MCM;FLExDUX4/+ combined with the similar velocity capacity allowed power to be maintained both in initial and critical conditions. This was evidenced in particular by the higher critical optimal velocity for the Acta1-MCM;FLExDUX4/+ (V_optc_ +32%, *p* = 0.003; Supplementary Figure S7D).

## 3. Discussion

This project aimed to precisely evaluate molecular, histological, and functional changes in an Acta-Cre/FLExDUX4 mouse model [25] at young ages. Our study focused on the TA and QUAD muscles, which differ in anatomical location, morphology, and function. The TA, situated on the anterior aspect of the lower leg, is primarily responsible for dorsiflexion of the foot and consists predominantly of fast-twitch type II fibres (IIb, IIx). Due to its accessibility, the TA is commonly utilised for ex vivo force production measurements and in therapeutic interventions for muscle disorders. In contrast, the QUAD is a large muscle group located on the anterior thigh, primarily responsible for knee extension, and plays a key role in posture maintenance and generating powerful movements. The QUAD has a more diverse fibre type composition (Type I, IIa, IIx, IIb) than the TA.

This bi-transgenic model developed by the Jones laboratory expresses tuneable levels of DUX4 when treated with different TMX dosing regimens [25,26], leading to different severities. TMX is well known as a selective oestrogen receptor modulator (SERM) that is used to treat diverse diseases, including breast cancer in both men and women [38]. Differential effects are produced by SERMs depending on the tissue and cell, acting as either agonists or oestrogen receptor antagonists. The prevention of bone loss and anti-inflammatory and fibrosis effects have been investigated for treating human disorders and muscle repair and the enhancement of muscle function. Several publications have reported beneficial effects of tamoxifen treatment in different mouse models of muscle disorders: in a mouse model of fukutin-related protein (FKRP) dystroglycanopathy, TMX and raloxifene treatment were shown to alleviate dystrophic phenotype and enhance muscle function in an FKRP mutant mouse [39]; in the mdx^5Cv^ mouse model of DMD, oral tamoxifen treatment conferred a markedly slower phenotype to the muscles, including reduced fibrosis in the diaphragm, increased force of the legs, and resistance to fatigue [40]; in mouse models of Bin1- and Dnm2-related centronuclear myopathies, exposure to tamoxifen resulted in improvement in muscle contractility associated with a rescue of histological alterations in these models [41]; in the Mtm1^−/y^ model of myotubular myopathy, TMX treatment resulted in increased survival and force production [42]. These data demonstrated that long systemic treatment with TMX can modify muscle physiology. In our study, we observed that low levels of TMX slightly increased the degenerative process, which was revealed by a more pronounced histological deterioration in the presence of TMX (Figure 1, Figure 2 and Figure 3). But tamoxifen also smoothed out male and female differences regarding the expression of target genes. The levels of *Wfdc3* and *Serpinb6* were always lower in females than in males in the absence of TMX. When mice were treated with TMX, there was no difference between males and females (Figure 5). Wfdc3 encodes a member of the WAP-type four-disulfide core (WFDC) domain family, which functions as a protease inhibitor, promoting the ERβ-mediated transcriptional repression of TGFBR1 in colorectal cancer [43], thus showing a link between WFDC3 and oestrogen receptor. Serpinb6, a protease inhibitor, has also been associated with oestrogen metabolism in the context of breast cancer. Two putative oestrogen response elements [44] have been identified in the region upstream of the Serpinb6 transcriptional start site, and Serpinb6 is regulated by 17β-oestradiol in oestrogen receptor-positive cancer cells [45]. It is interesting to note that gender differences in disease severity in FSHD patients have been previously observed. There is a higher proportion of women among asymptomatic gene carriers [46], and FSHD1 affects males more severely and frequently [47].

Our study investigated several histological and molecular changes that could be used as markers of therapeutic efficacy in the FLExDUX4/+ and ACTA1-MCM/+;FLExDUX4/+ models before 20 weeks. We observed that both the TA and QUAD muscles exhibited a progressive degenerative process, characterised by changes in muscle architecture, macrophage infiltration, and regenerating myofibers, confirming the results of a previous publication [29]. The QUAD exhibited greater susceptibility to pathology compared to the TA. Several hypotheses can be proposed, including embryological and developmental factors, vascular and metabolic factors, muscle fibre composition, and varying susceptibility to DUX4 expression. Furthermore, spontaneous recombination events leading to DUX4 expression without TMX were not uniformly distributed across all the muscle groups. Notably, the QUAD demonstrated twice the rate of spontaneous recombination compared to the TA [26], which may have contributed to the observed differences between these muscles. Intriguingly, the QUAD muscles showed a very high expression of fibrosis-related genes at 12 weeks. This could have resulted from DUX4 expression a few weeks before, as suggested by the increase in the DUX4 network genes at 8–12 weeks. This is consistent with previously published data [48], showing that a doxycycline pulse induction protocol in iDUX4pA HSA mice leads to an up-regulation of fibrosis-related genes. However, the precise mechanism by which DUX4 expression can be initiated prematurely and independently of TMX remains unidentified.

At molecular levels, the FLExDUX4/+ animals were previously described to express DUX4 mRNA, which is not translated into the DUX4 protein [25,26]. No difference in *DUX4-fl* mRNA levels was reported between the FLExD/+ and the Cre-FLeX with or without TMX [26]. In our study, we were able to detect an increase in *DUX4* mRNA in the Acta1-MCM;FLExDUX4/+ animals compared to the FLExDUX4/+ ones (Figure 4). The use of the DUX4-UTR primer set we previously published [4] allowed the amplification of the DUX4-FL2 isoform, retaining DUX4 intron 1. The DUX4-FL1 isoform, in which introns 1 and 2 are spliced, was not visible on the gel. This agrees with what is seen in human cells and biopsies, where *DUX4* isoform FL1 is barely observed. Interestingly and according to Nunes et al. [29], the Acta1-MCM;FLExDUX4/+ mice displayed higher DUX4-fl expression with age compared with ACTA1-MCM/+ controls, arguing against DUX4 immediate toxicity when expressed at low levels [29]. The *DUX4* mRNA was translated into a DUX4 protein in the Acta1-MCM;FLExDUX4/+ animals as four genes downstream of DUX4 (*Wfdc3*, *Agtr2*, *Serpinb6*, and *Ilvbl*) were expressed in the Acta1-MCM;FLExDUX4/+ animals only. Interestingly, the expression profiles of these four genes were very different from each other. We observed a strong correlation between *Wfdc3* expression and the age of the animals, while *Agtr2* expression showed an inverted correlation (Figure 5). *Serpinb6* and *Ilvbl* did not vary with age. This highlights the importance of external factors that may influence the expression of the genes downstream of DUX4. This may be significant because in clinical trials targeting DUX4, DUX4 levels are not used as an outcome measure; instead, a DUX4 composite score is used. As DUX4 regulates overlapped and distinct groups of genes and pathways in human and mouse cells [49], different genes are used in mouse and human studies. The DUX4 composite score used in clinical trials may vary from one study/trial to another. It may be influenced by several factors, including age, gender, muscle, the presence of fibrotic tissue, fat infiltration, inflammation, etc. Moreover, DUX4-activated genes include both direct and indirect targets of DUX4, with different activation timelines. Most of the DUX4-mediated dysregulation of transcription appears to be secondary, which may also indicate that several factors beyond DUX4 may play a role in their activation [50]. For example, among the genes proposed to be included in the DUX4 composite score in different studies, ZSCAN4, MND3L2, LEUTX, or TRIM43 contain a DUX4 binding site within 5 kb upstream or downstream of a transcriptional start site, but CCNA1 and SLC2A3 do not [50]. Finally, the mRNA half-life may also significantly influence this DUX4 composite score. These results highlight the need to develop new biomarkers for disease severity and/or therapeutic efficacy [51].

So far, several force tests have been proposed in the literature such as the grip strength test to assess the limb strength and in vitro or in situ measurement of maximum muscle force-producing capacity. In situ measurements have advantages over in vitro measures since the muscle is maintained in its natural environment with normal vascularisation and innervation. It has been shown that the results obtained in characterising force production differ between in situ and ex vivo measurements. In situ set-ups are closer to physiological muscle function (e.g., temperature, O_2_ supply) and probably more representative of real muscle function. In this study, we showed that the maximum force of the TA was ~40% lower in Acta1-MCM;FLExDUX4/+ animals. This is in line with previous publications showing that the EDLs of Acta1-MCM;FLExDUX4/+ animals are weaker than those of ACTA1-MCM/+ animals [25,26]. We also observed that this weakness is partly due to a loss of muscle mass since the specific force was not different at high stimulation frequencies (≥60 Hz).

However, mechanisms other than mass exist, since a right shift in the force–frequency relationship has been demonstrated in Acta1-MCM;FLExDUX4/+ mice (e.g., lower P0s 20 Hz and P0s 40 Hz and lower ϕ). A greater stimulation frequency is necessary to generate the same level of relative force, which can be attributed to a lower calcium sensitivity and has been associated with the muscle fibre phenotype. Typically, faster twitches show lower calcium sensitivity [52] and a right-shifted force–frequency relationship [53]. The lower specific force at low frequencies observed in Acta1-MCM;FLExDUX4/+ mice could therefore be linked to a faster muscle phenotype as previously observed in these mice. Other results from the functional muscle evaluations are also consistent with this hypothesis. For instance, the higher relative rate of force development (i.e., lower τ_RFD_) observed in Acta1-MCM;FLExDUX4/+ would be in line with a greater proportion of fast twitches [54]. Consequently, increasing the muscle mass without addressing the primary cause of the disease, namely the expression of DUX4, will only partially compensate for the loss of muscle force.

The previously discussed results relate to force evaluation without muscle shortening, which does not reflect the muscle contraction modalities in ecological conditions. Characterisation of muscle capacities from the force–velocity–endurance (FoVE) model that we developed allows a better description of the muscle function of the ACTA1-MCM/+ and Acta1-MCM;FLExDUX4/+ transgenic mice. Mainly, this model enables the analysis of the force, velocity, and power components of muscular mechanical properties both in fresh and fatigued states. The FoVE evaluation showed similar results to traditional evaluations on the force component (i.e., a lower maximal absolute, but not relative, force capacity for fresh and fatigued states). However, the absolute maximum power (in mW) was not statistically different between the groups. The non-significant lower curvature C_i_ and higher velocity V_0i_ combined seem to have maintained the muscle power capacity of the Acta1-MCM;FLExDUX4/+ mice. The curvature reflects changes in the rates of cross-bridge attachment to, and detachment from, the actin filaments. The lower curvature mechanically brings an advantage in force production at intermediate speed in the Acta1-MCM;FLExDUX4/+ animals, allowing maintenance of the power capacities. In other words, for a given force production, the muscle can produce it with a higher shortening velocity, leading to more power production. This may be one reason why the muscles of the Acta1-MCM;FLExDUX4/+ animals showed less of a power decrease during the test.

As the curvature of the force–velocity relationship is known to be lower for fast muscles [55], this is an additional argument, along with those mentioned above, for potential evolution of the Acta1-MCM;FLExDUX4/+ phenotype towards faster fibres. This is even more marked under fatigue conditions, where the Cc of Acta1-MCM;FLExDUX4/+ is almost null. Under fatigue conditions, the difference in curvature between fast and slow muscles becomes even more marked [56], as observed in the present study. Taken together (right-shifted force–frequency relationship, lower τ_RFD_, lower force–velocity curvature under fatigue condition), the mechanical evaluation of the Acta1-MCM;FLExDUX4/+ muscles are in line with a potential phenotypic modification of the muscle towards a faster profile. These adaptations could make it possible to compensate for the associated loss of mass and force by maintaining the velocity production and a low curvature of the force–velocity relationship to maintain power production capacities.

Surprisingly, although the muscles of the Acta1-MCM;FLExDUX4/+ mice tended to produce less force at the end of the test, they seemed to be relatively more resistant to fatigue development than those of Acta1-MCM/+ mice (higher τ and lower relative force and power decrease compared to initial capacity). Among the different possibilities explaining these results, we can note the slowing down of the speed of conduction of the action potential on the surface of the fibre, the modification of the transmission of the signal from the T tubules to the sarcoplasmic reticulum, the quantitative and qualitative variations in the release of calcium from the sarcoplasmic reticulum, changes in the formation of actin–myosin bridges, and a deficiency in ATP resynthesis [57]. The capillary density could also have been modified, resulting in a change in convective O_2_ delivery.

## 4. Materials and Methods

### 4.1. Animal Housing, Breeding, and Genotyping

The mice were bred in the Biological Services Unit of the Great Ormond Street Institute of Child Health and UCL under the Animals (Scientific Procedures) Act 1986 and under licence PB49D7D47 from the Home Office. The UK and European guidelines (Directive 2010/63/UE of the European Parliament and of the Council) were followed in all experiments. The FLExDUX4 (B6(Cg)-Gt(ROSA) 26Sortm1.1(DUX4*)Plj/J; RRID:MGI:5707284) and ACTA1-MCM (B6.Cg-Tg(ACTA1-cre/Esr1)2Kesr/J; RRID:IMSR_JAX:031934) mice were purchased from the Jackson Laboratory (#028710, #025750 respectively). Hemizygous ACTA1-MCM/+ and homozygous FLExDUX4 mice were crossed to generate the Acta1-MCM;FLExDUX4/+ mice. The genotyping is described elsewhere [58]. In some cases, to increase DUX4 expression, mice were treated with tamoxifen (TMX) via intraperitoneal injection and weighed 2–3 times weekly. Males received tamoxifen at 2.5 mg/kg/week for 4 weeks, and females received a biweekly injection of 2.5 mg/kg (our preliminary experiments have indicated that females are more susceptible to tamoxifen than males). TMX was injected into 16-week old mice that were sacrificed 4 weeks later. TMX was prepared as previously described [25]. Muscles were harvested from cervically dislocated animals and frozen in liquid nitrogen or liquid nitrogen-cooled isopentane for further analysis.

### 4.2. Histological and Immunofluorescence Analysis

Histological and immunofluorescence analyses were performed on 10 μm transverse cryosections. Sections stored at −80 °C were dried at room temperature for 30 min. Cryosections were stained with haematoxylin for 8 min, followed by a 4 min incubation with eosin, before being dehydrated by increasing ethanol concentration. Sections stained with haematoxylin were also used to count the number of centrally located nuclei. The protocol followed for the CD68 staining is described elsewhere [21]. For embryonic Myosin Heavy Chain (eMyHC) and laminin immunostaining, sections were incubated in a PBS solution containing 1% BSA, 1% goat serum, 1:25 diluted MOM blocking reagent (Vector Laboratories, Newark, CA, USA), and 0.1% triton X-100 for 30 min and then incubated with 1:400 diluted polyclonal Rabbit Anti Laminin (#Z0097, DAKO, Santa Clara, CA, USA) for 1 h at room temperature. After being washed, sections were incubated with 1:100 diluted mouse anti-embryogenic MyHC (#BF-G6, DSHB, Iowa City, IA, USA) in PBS, 1% BSA, and 1% goat serum at 4 °C overnight. After 3 washes, sections were incubated with the secondary antibodies, goat anti-rabbit IgG Alexa 488 (#A11034, Thermo Fisher Scientific, Loughborough, UK; 1/400) and goat anti-mouse IgG Alexa 568 (#A11004, Life Technologies, Paisley, UK; 1/400) for laminin and eMyHC, respectively. Nuclei were stained with Hoechst (#H21492, Thermo Fisher Scientific, Loughborough, UK, 1/2000) for 10 min. The sections for picrosirius red staining were hydrated by decreasing the ethanol concentration, washed with water, and fixed with 4% paraformaldehyde (PAF) for 30 min. Sections were then stained in haematoxylin for 8 min and in picrosirius red solution (#AB150681; Abcam, Cambridge, UK) for 1 h. Sections were washed quickly in acetic acid and dehydrated in a 100% ethanol bath for 3 min each, cleared with xylene for 30 min, and mounted. Pictures were acquired using an EVOS™ FL Auto 2 Imaging System (Thermo Fisher Scientific, Loughborough, UK) and 20× objectives. Sections were scanned entirely, and pictures were analysed using Image J2 Version 2.14.0/1.54f software. The number of fibres with centrally located nuclei (CLN) was counted and compared to the total number of fibres within 5 randomly selected squares over each section. For CD68 and eMyHC, the total number of CD68 and eMyHC-positive fibres was counted and compared to the total number of fibres in the entire muscle. Statistical analyses were carried out using GraphPad Prism 9.

Fibrosis quantification was performed on picrosirius red-stained images using ImageJ software. The red colour channel was isolated and converted to grayscale, with pixel intensity values ranging from 0 (black) to 255 (white). A specific white threshold was established for each image to set the counting limit. The total number of pixels within each image was counted, and the percentage of pixels corresponding to the red channel, relative to the total pixel count, was calculated to represent the fibrosis percentage.

### 4.3. RNA Extraction PCR and Quantitative PCR

Trizol was used to extract total RNA from muscles frozen in liquid nitrogen. In total, 1 μg of total RNA was used to perform reverse transcription (Transcriptor First-Strand cDNA Synthesis Kit, Roche Diagnostics Limited, Burgess Hill, UK) in a final volume of 10 μL. PCR was performed with 1 μL of RT product and 1 μL of each forward and reverse primer at 20 pmol/μL in a final volume of 25 μL. The primers used in this study are listed in Table 1. Thermal cycling conditions were 94 °C for 5 min and then 36 cycles at 94 °C for 30 s and 62 °C for 45 s [4], with primers allowing the amplification of the different DUX4 isoforms. Quantitative PCR was realised as previously described [59]. Briefly, qPCRs were performed on a LightCycler 480 Real-Time PCR System (Roche Diagnostics Limited, Burgess Hill, UK) in a final volume of 9 µL with 0.2 μL of RT product, 0.4 µM each of forward and reverse primer [21,48], and 4.5 μL of SYBRGreen Mastermix (Roche Diagnostics Limited, Burgess Hill, UK). Gapdh was used a normaliser in qPCR experiments.

### 4.4. Force Test

The distal tendon of the TA muscle (from males only) was attached to the force-position transducer apparatus (Aurora Scientific, Aurora, ON, Canada). At the same time, the mouse’s knee was anchored, and the sciatic nerve was stimulated. For subsequent tests, isometric contractions were realised at the optimum muscle length (L0) The protocol described in detail below included an assessment of the force–frequency and force–velocity–endurance relationships.

For the force–frequency assessment, maximum force productions in response to tetanic stimulations (P0) were recorded for pulse frequencies from 20 to 120 Hz every 20 Hz with a train duration of 500 ms, and 3 min was allowed between each contraction. The force–frequency relationship was fitted with a SIGMOID function to obtain the P0 plateau and φ, the characteristic frequency at which ≈63% of the P0 plateau was reached. In addition, an isometric fatigue protocol was performed with a continuous stimulation frequency of 60 Hz for 2 min.

Afterward, for the initial force–velocity capacity (before fatigue), 12 shortening contractions were performed on a 1.5 mm range (~25% of muscle length) at velocities regularly spaced between 2.4 and 30 mm s^−1^ with a 100 Hz pulse frequency and interspersed with 1 min of recovery. Hill’s hyperbolic function (Equation (1); [60]) was fitted with the maximum force measured during each of these contractions at different velocities to obtain the maximum force at null velocity (F_0i_), the maximum velocity at which force production is still possible (V_0i_), and curvature of the relationship (C_i_). From these parameters, the maximum power produced (P_maxi_) and the velocity at which the power was maximised (V_opti_) were calculated.

(1) $$F\left(V\right)=F_{0}\cdot \left(\frac{V_{0}-V}{V_{0}+C\cdot V}\right)$$

After a 5 min recovery period, force–velocity–endurance capacities were assessed during a fatiguing 180 s exercise. The protocol consisted of repeating shortening contractions performed at different velocities, with a 100 Hz pulse frequency and 0.5 s of passive lengthening (i.e., returning to the starting position before the next contraction). During 180 s, the imposed shortening velocity followed a sinusoidal function between 2 and 15 mm s^−1^ and a 30 s period. The maximum force measured during each contraction was used to adjust the F(v,t) function based on Hill’s equation and the exponential decrease in contractile capacity during prolonged maximum effort (Equation (2)). This made it possible to obtain the force–velocity parameters under extreme fatigue conditions (F_0c_, V_0c_, C_c_, P_maxc_, and V_optc_) and the time constant τ describing the decrease in capacity over time (at τ s, 63% of the final force–velocity capacity decrease was reached). All force and power indices were expressed both as absolute values and values normalised to the muscle mass.

(2) $$F\left(V,t\right)=F_{c}\left(V\right)+\left(F_{i}\left(V\right)-F_{c}\left(V\right)\right)\cdot e^{\left(-\frac{t}{\tau }\right)}$$

## 5. Conclusions

Our study has established a range of histological, molecular, and functional assays that can be routinely employed in the Acta1-MCM; FLExDUX4/+ mouse model to assess the efficacy of therapeutic interventions. Notably, we have developed comprehensive force measurement protocols that more accurately mimic everyday physiological movements and can be applied to young animals (12–13 weeks of age) without tamoxifen induction. Further investigations are required to characterise this model’s additional parameters, such as oxidative stress, to expand our understanding of disease mechanisms and therapeutic outcomes.

Limitations of the study: It should be noted that the functional muscle capacity assessment involved two fatiguing evaluations. Although significant recovery was observed between the 2 min 60 Hz stimulation protocol and the FoVE protocol, maximum capacity was still reduced at the start of the second protocol. These methodological choices have been made to present conventional evaluations (2 min 60 Hz tetanos) and innovative FoVE approaches. Although the initial absolute values of force and velocity are probably underestimated in the FoVE results, this should not affect the relative comparisons between the groups and the resulting interpretations.

## Figures and Tables

**Table 1:** List of the primers used in this study.

Gene Symbol	Accession Number	Name	Primer Sequence (5′–3′)	Amplicon Size (bp)
*Gapdh*	NM_001289726.1	Gapdh_F	TTGTGATGGGTGTGAACCAC	283
		Gapdh_R	TTCAGCTCTGGGATGACCTT	
*Wfdc3*	NM_001418735.1	Wfdc3-F	GGTAGCTGCAGGAGAGCACG	94
		Wfdc3-R	CTGGGGACAGGATTCGTCTC	
*Ilvbl*	NM_173751	Ilvbl_F	AGGAGCTTCGGAAAGCTGAC	105
		Ilvbl_R	CCACCTGCTGTAACACCCAT	
*Serpinb6c*	NM_148942	Serpinb6c_F	CAGTCCCGACAGCACATCAA	178
		Serpinb6c_R	TGAATGGCATCTCCCTGGTG	
*Agtr2*	NM_007429.5	Agtr2_F	TTTTAAGGAGTGCATGCGGG	159
		Agtr2_R	GGACGGCTGCTGGTAATGT	
*Pdgfra*	NM_011058.3	Pdgfra-F	AAAATTGTGTCCACCGGGACC	194
		Pdgfra-R	ACTCAGCGTGGTGTAGAGGT	
*Col1a1*	NM_007742.4	Col1a-F	GAGCGGAGAGTACTGGATCG	204
		Col1a-R	TACTCGAACGGGAATCCATC	
*Col3a1*	NM_009930.2	Col3a1-F	TGGTCCTCAGGGTGTAAAGG	221
		Col3a1-R	GTCCAGCATCACCTTTTGGT	
*DUX4-UTR*	HQ266761	DUX4-UTR_F	AGGCGCAACCTCTCCTAGAAAC	368 and 504
		DUX4-UTR_R	TCCAGGAGATGTAACTCTAATCCA	
*Psma2*	NM_008944.2	Psma2-F	AGAGCGCGGTTACAGCTTC	193
		Psma2-R	CTCCACCTTGTGAACACTCCTT	

## Data Availability

Data is contained within the article and Supplementary Materials.

## Supplementary Materials

The following supporting information can be downloaded at: https://www.mdpi.com/article/10.3390/ijms252111377/s1.
