# Supplementary material for: Molecular, Histological, and Functional Changes in Acta1-MCM;FLExDUX4/+ Mice
Source: Int J Mol Sci. 2024 Oct 23;25(21):11377. doi: 10.3390/ijms252111377 (PMC11545788; doi:10.3390/ijms252111377)
Supplement: Supplementary file 1 [file ijms-25-11377-s001.zip › ijms-3225398-supplementary.pdf]

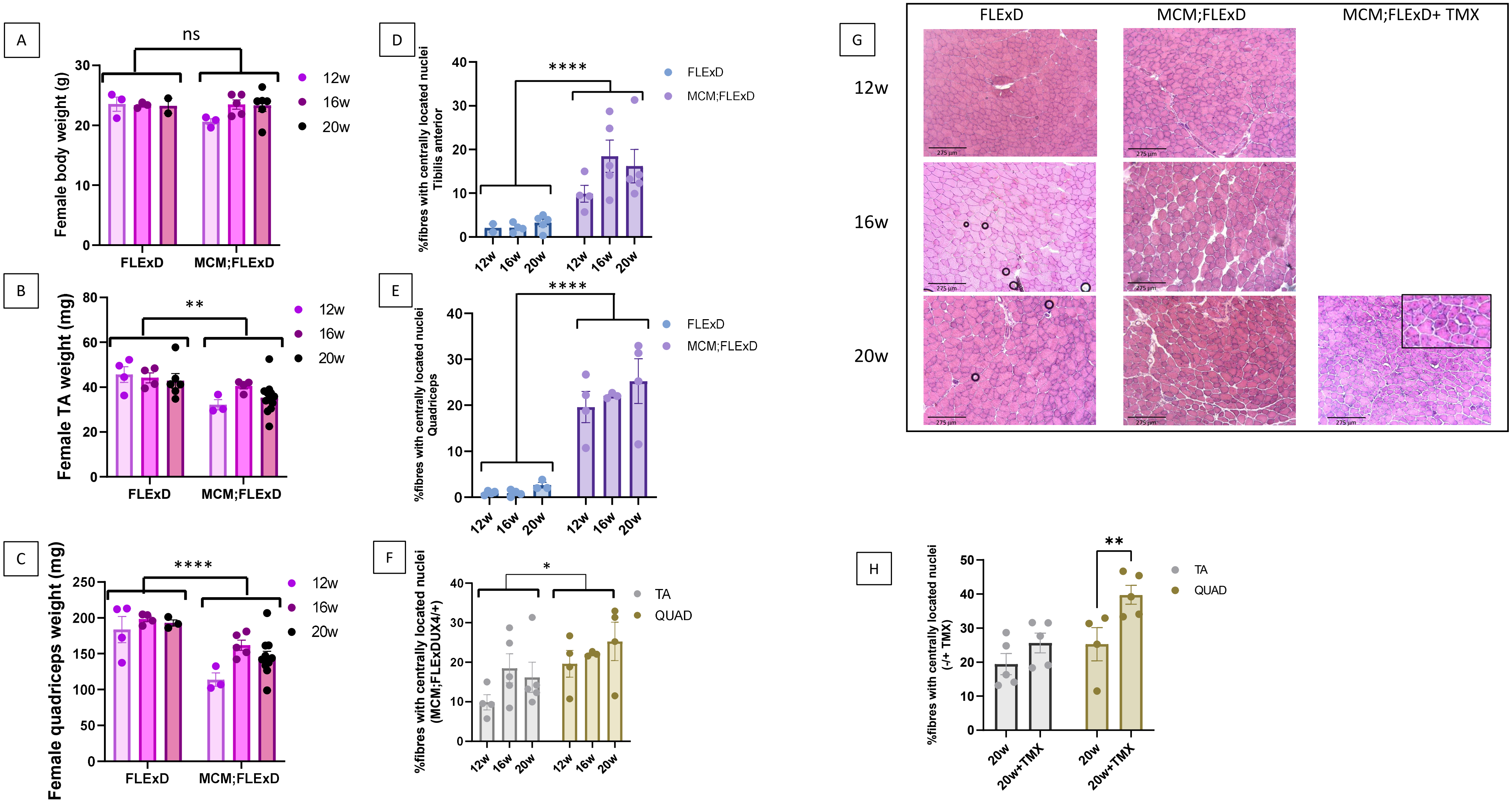

A
ns
Female body weight (g)
0
10
20
30
FLExD
MCM;FLExD
12w
16w
20w
B
**
Female TA weight (mg)
0
20
40
60
80
FLExD
MCM;FLExD
12w
16w
20w
C
****
Female quadriceps weight (mg)
0
50
100
150
200
250
FLExD
MCM;FLExD
12w
16w
20w
D
****
%fibres with centrally located nuclei
Tibilis anterior
0
10
20
30
40
12w
16w
20w
12w
16w
20w
FLExD
MCM;FLExD
E
****
%fibres with centrally located nuclei
Quadriceps
0
10
20
30
40
12w
16w
20w
12w
16w
20w
FLExD
MCM;FLExD
F
*
%fibres with centrally located nuclei
(MCM;FLExDUX4/+)
0
10
20
30
40
12w
16w
20w
12w
16w
20w
TA
QUAD
G
FLExD
MCM;FLExD
MCM;FLExD+ TMX
12w
16w
20w
H
**
%fibres with centrally located nuclei
(-/+ TMX)
0
10
20
30
40
50
20w
20w+TMX
20w
20w+TMX
TA
QUAD

Supplementary Figure S1: Acta1-MCM;FLExDUX4/+ females present a slow dystrophic process.
The total body (A), tibialis anterior weight (B), and quadriceps weight (C), and the percentage of centrally located nuclei in the tibialis anterior (D and F) and the quadriceps (E and F) were measured at 12, 16 and 20 weeks in females. In some animals, TMX (2.5 mg/kg weekly for 4 weeks) was injected (H).
Error bars indicate the mean of SEM of 3-5 biological replicates. p-values were calculated using GraphPad Prism9, 2-way ANOVA followed by a Fisher's LSD post hoc test. *$p < 0.05$; **$p<0.01$; ***$p<0.001$; ****$p<0.0001$.

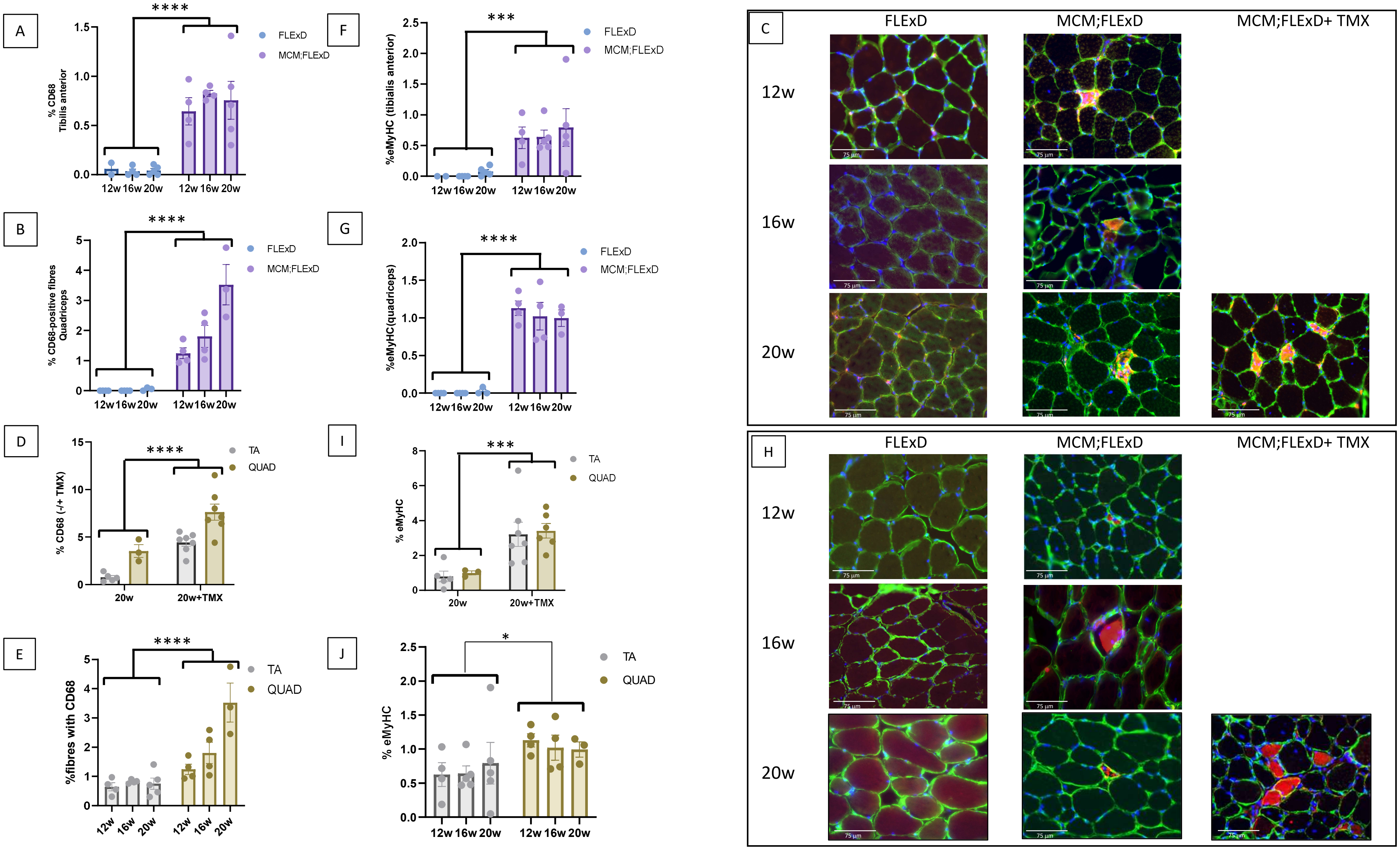

A
% CD68 Tibilis anterior
FLExD
MCM;FLExD
12w 16w 20w 12w 16w 20w
B
% CD68-positive fibres Quadriceps
D
% CD68 (-/+ TMX)
TA
QUAD
20w
20w+TMX
E
%fibres with CD68
F
%eMyHC (tibialis anterior)
G
%eMyHC(quadriceps)
I
% eMyHC
J
% eMyHC
C
FLExD
MCM;FLExD
MCM;FLExD+ TMX
12w
16w
20w
H

Supplementary Figure S2: Acta1-MCM;FLExDUX4/+ animals present inflammation and regenerative fibres.

The percentage of fibres positive for CD68 staining (A-E) and the percentage of eMyHC positive fibres (F-J) were measured in the tibialis anterior (A, F) and the quadriceps (B, G) at 12, 16, and 20 weeks in FLExDUX4/+ and ACTA1-MCM/+;FLExDUX4/+ (MCM;FLExD) males. In some animals, TMX (2.5 mg/kg weekly for 4 weeks) was injected (C,D, H, I). The number of fibres positive for CD68 staining or eMyHC was compared in the TA and QUAD muscles (E&J, respectively). Error bars indicate the mean of SEM of 3-5 biological replicates. p values were calculated using GraphPad Prism9, 2-way ANOVA followed by a Fisher's LSD post hoc test. $*p < 0.05$; $**p<0.01$; $***p<0.001$; $****p<0.0001$.; TMX: tamoxifen.

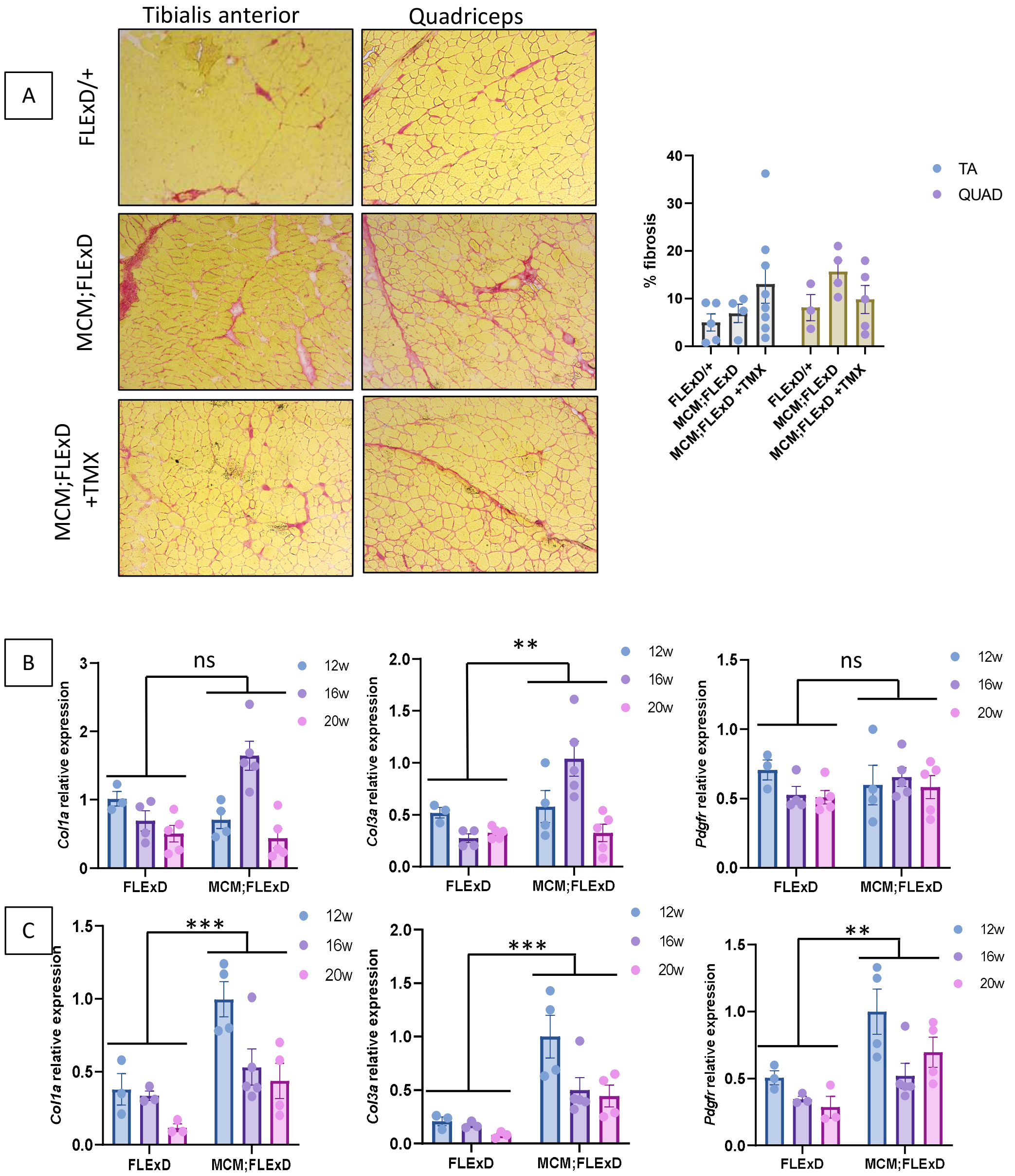

A
Tibialis anterior
Quadriceps
FLExD/+
MCM;FLExD
MCM;FLExD +TMX
% fibrosis
TA
QUAD
FLExD/+
MCM;FLExD
MCM;FLExD +TMX
B
C
Col1a relative expression
Col3a relative expression
Pdgfr relative expression
ns
**
***
12w
16w
20w
FLExD
MCM;FLExD

Supplementary Figure S3: Acta1-MCM;FLExDUX4/+ present fibrotic infiltration.

Acta1-MCM;FLExDUX4/+ or FLExDUX4/+ females were treated with or without tamoxifen. Picrosirius red was used to stain collagen on the tibialis anterior (upper panel) and quadriceps (lower panel) sections (A) in animals killed at the age of 20 weeks. The expression of several genes involved in fibrosis was investigated in the tibialis anterior (B) and quadriceps (C) in the females at the age of 12, 16 or 20 weeks. Error bars indicate the mean of SEM of 3-5 biological replicates. p values were calculated using GraphPad Prism9, 2-way ANOVA followed by a Fisher's LSD post hoc test. *p < 0.05; **p<0.01; ***p<0.001; ****p<0.0001.; TMX: tamoxifen.

A

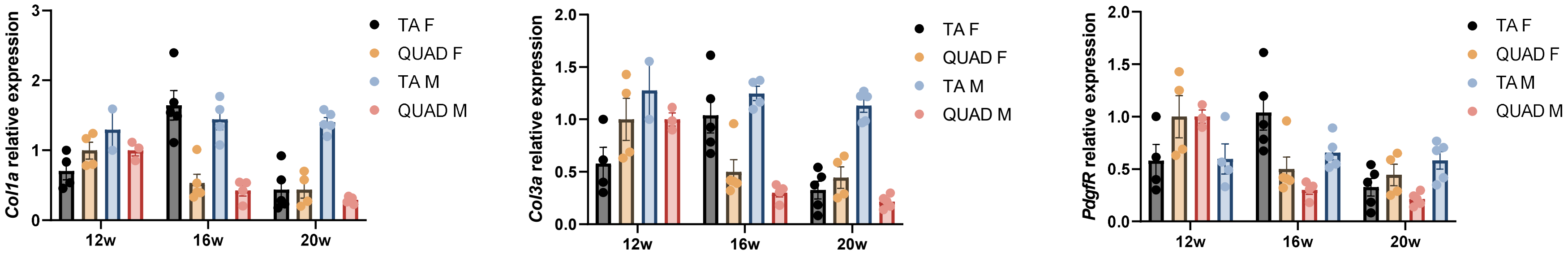

Source of Variation	% of total vari	P value	P value summar
age	11.09	0.0002	***
muscle	2.857	0.0256	*
gender	24.75	<0.0001	****
age x muscle	5.668	0.0089	**
age x gender	14.76	<0.0001	****
muscle x gender	6.033	0.0017	**
age x muscle x gende	6.059	0.0067	**

Source of Variation	% of total variation	P value	P value summary
age	15.63	<0.0001	****
muscle	5.761	0.0045	**
gender	16.10	<0.0001	****
age x muscle	2.942	0.1118	ns
age x gender	12.85	0.0003	***
muscle x gender	16.09	<0.0001	****
age x muscle x gende	2.459	0.1575	ns

Source of Variation	% of total variation	P value	P value summary
age	3.187	0.1276	ns
Muscle	12.09	0.0002	***
gender	9.043	0.0012	**
age x Muscle	0.5442	0.6923	ns
age x gender	13.06	0.0007	***
Muscle x gender	22.00	<0.0001	****
age x Muscle x gender	2.268	0.2259	ns

B

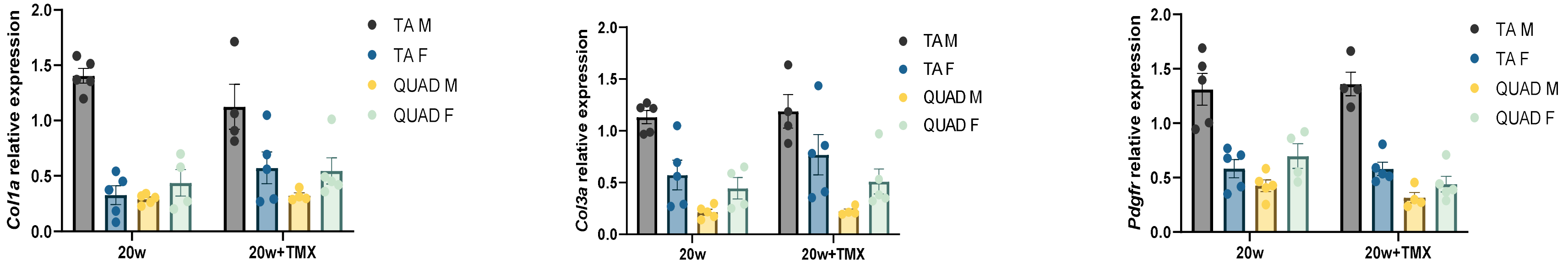

Source of Variation	% of total variation	P value	P value summary
-/+ TMX	0.09293	0.7309	ns
muscle	26.65	<0.0001	****
Gender	12.59	0.0004	***
-/+ TMX x muscle	0.2366	0.5838	ns
-/+ TMX x Gender	2.872	0.0634	ns
muscle x Gender	31.74	<0.0001	****
-/+ TMX x muscle x Ger	1.595	0.1609	ns

Source of Variation	% of total variation	P value	P value summary
-/+ TMX	0.9503	0.3572	ns
muscle	46.30	<0.0001	****
Gender	1.917	0.1943	ns
-/+ TMX x muscle	0.2761	0.6178	ns
-/+ TMX x Gender	0.3420	0.5789	ns
muscle x Gender	20.14	0.0002	***
-/+ TMX x muscle x Gend	0.06472	0.8088	ns

Source of Variation	% of total variation	P value	P value summary
-/+ TMX	0.9641	0.2241	ns
muscle	35.45	<0.0001	****
gender	11.43	0.0002	***
-/+ TMX x muscle	1.594	0.1211	ns
-/+ TMX x gender	0.3577	0.4553	ns
muscle x gender	33.54	<0.0001	****
-/+ TMX x muscle x gende	0.08550	0.7141	ns

Supplementary Figure S4: The expression of Col1A, Col3A and Pdgfr were compared in the TA or QUA muscles isolated from MCM;FLExDUX4/+ males or females at the age of 12, 16 or 20 weeks (A). Some animals were injected with tamoxifen (TMX) at the age of 16 weeks and muscles were harvested at the age of 20 weeks and compared to age-matched mice (B).
Error bars indicate the mean of SEM of 3-5 biological replicates. p values were calculated using GraphPad Prism9, 3-way ANOVA followed by a Fisher’s LSD post hoc test.

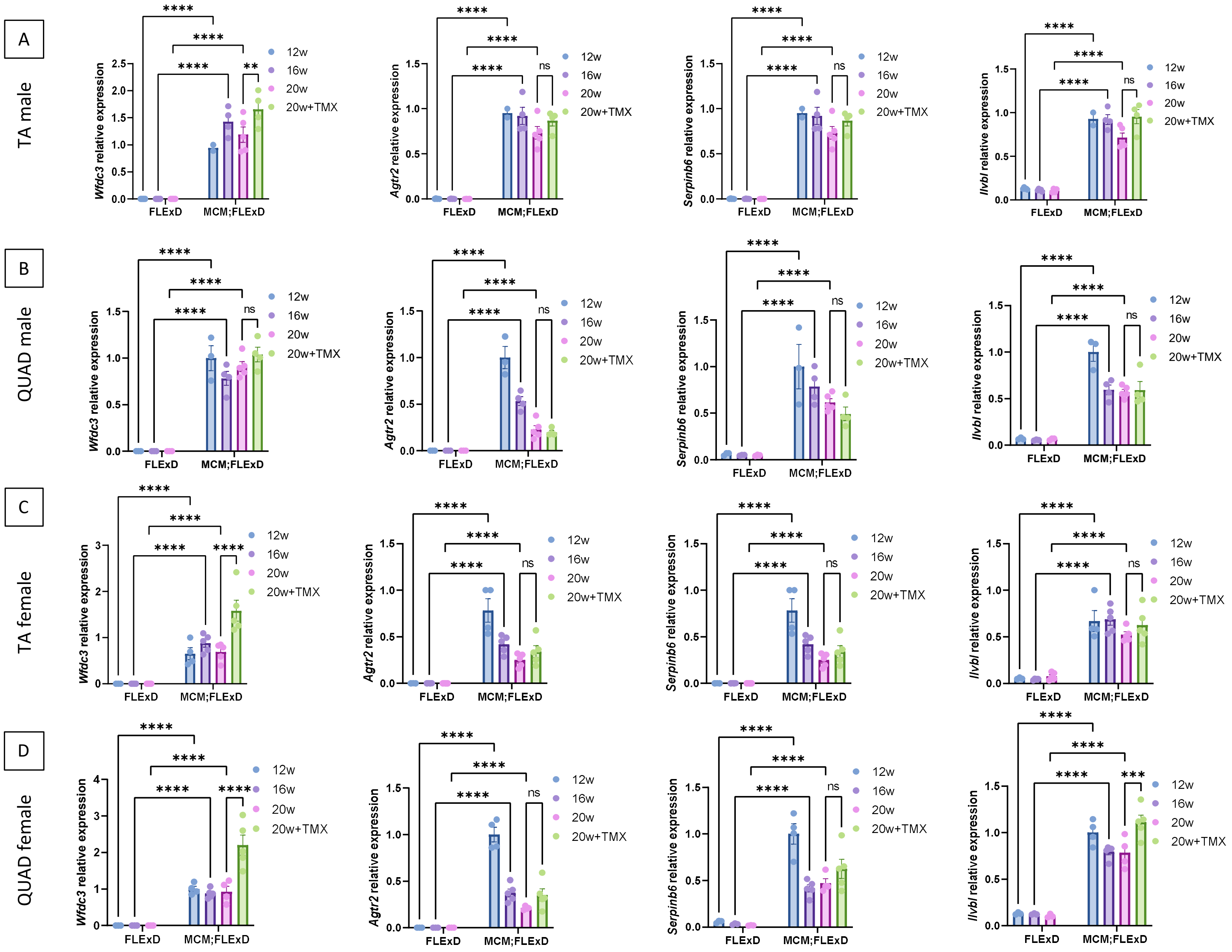

Supp Figure 5

Supplementary Figure S5: expression of genes downstream of DUX4 in males (A&B) and females (C&D). RT-PCR for Wfdc3, Agtr2, Serpinb6 and Ilvbl in the tibialis anterior (A&C) and the quadriceps (B&D) muscles of FLExDUX4/+ and MCM;FLExDUX4/+ animals at the age of 12, 16 or 20 weeks. Some animals were injected with tamoxifen at the age of 16 weeks and muscles were harvested at the age of 20 weeks and compared to age-matched mice. Error bars indicate the mean of SEM of 3-5 biological replicates. p values were calculated using GraphPad Prism9, 2-way ANOVA followed by a Fisher's post hoc test. *p < 0.05; **p<0.01; ***p<0.001; ****p<0.0001. TMX: tamoxifen.

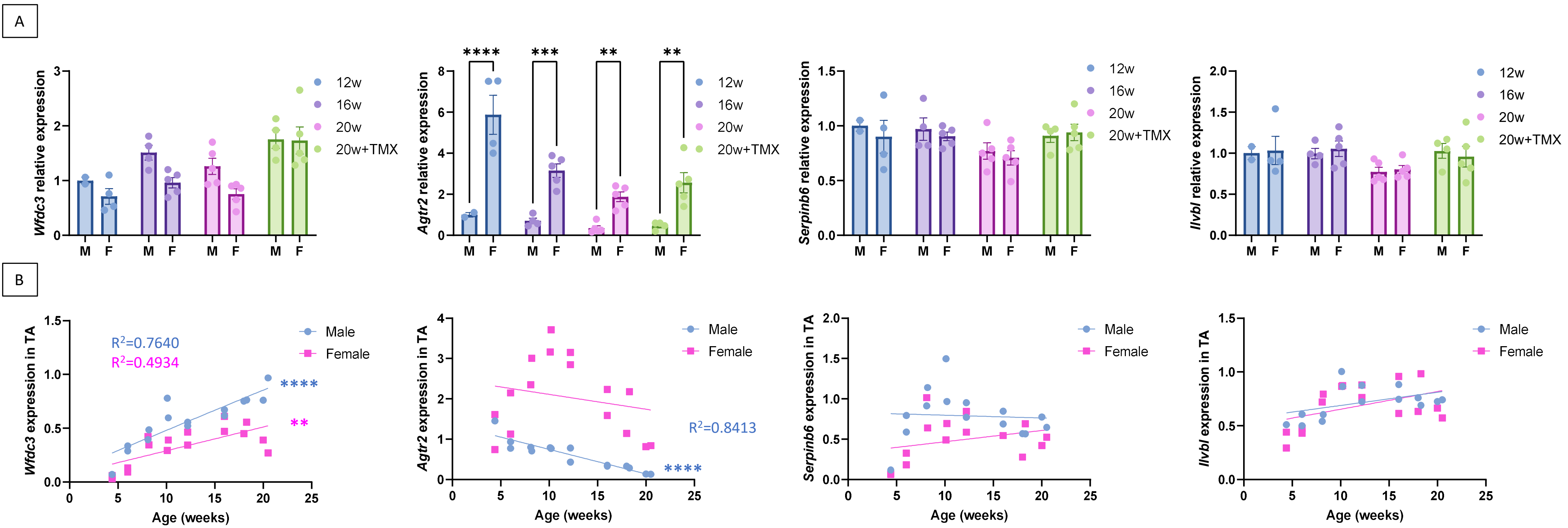
A
Wfdc3 relative expression
Agtr2 relative expression
Serpinb6 relative expression
Ilvbl relative expression
****
***
**
**
12w
16w
20w
20w+TMX
M F M F M F M F
B
Wfdc3 expression in TA
R^2=0.7640
R^2=0.4934
****
**
Agtr2 expression in TA
R^2=0.8413
****
Serpinb6 expression in TA
Ilvbl expression in TA
Male
Female
Age (weeks)

Supplementary Figure S6: Expression of the genes downstream of DUX4 in the MCM;FLExDUX4/+ tibialis anterior muscles

The expression *Wfdc3, Agtr2, Serpinb6* and *Ilvbl* was compared in the tibialis anterior of MCM;FLExDUX4/+, at the age of 12, 16 or 20 weeks, and in the absence or presence of tamoxifen (A). Error bars indicate mean of SEM of 3-5 biological replicates. P-values were calculated using Graphpad Prism9, 2-way ANOVA followed by a Fisher's LSD post hoc test. In B and C, the levels of *Wfdc3, Agtr2, Serpinb6* and *Ilvbl* were followed from 4 to 20 weeks of MCM;FLExDUX4/+ males or female. P-values were calculated using Graphpad Prism9 after a simple linear regression analysis. A low P-value (< 0.05) means that the slope is likely not to equal zero. R^2 are indicated on each graph p<0.05. TMX: tamoxifen ; M : male ; F : female. p < 0.05; **p<0.01; ***p<0.001; ****p<0.0001.

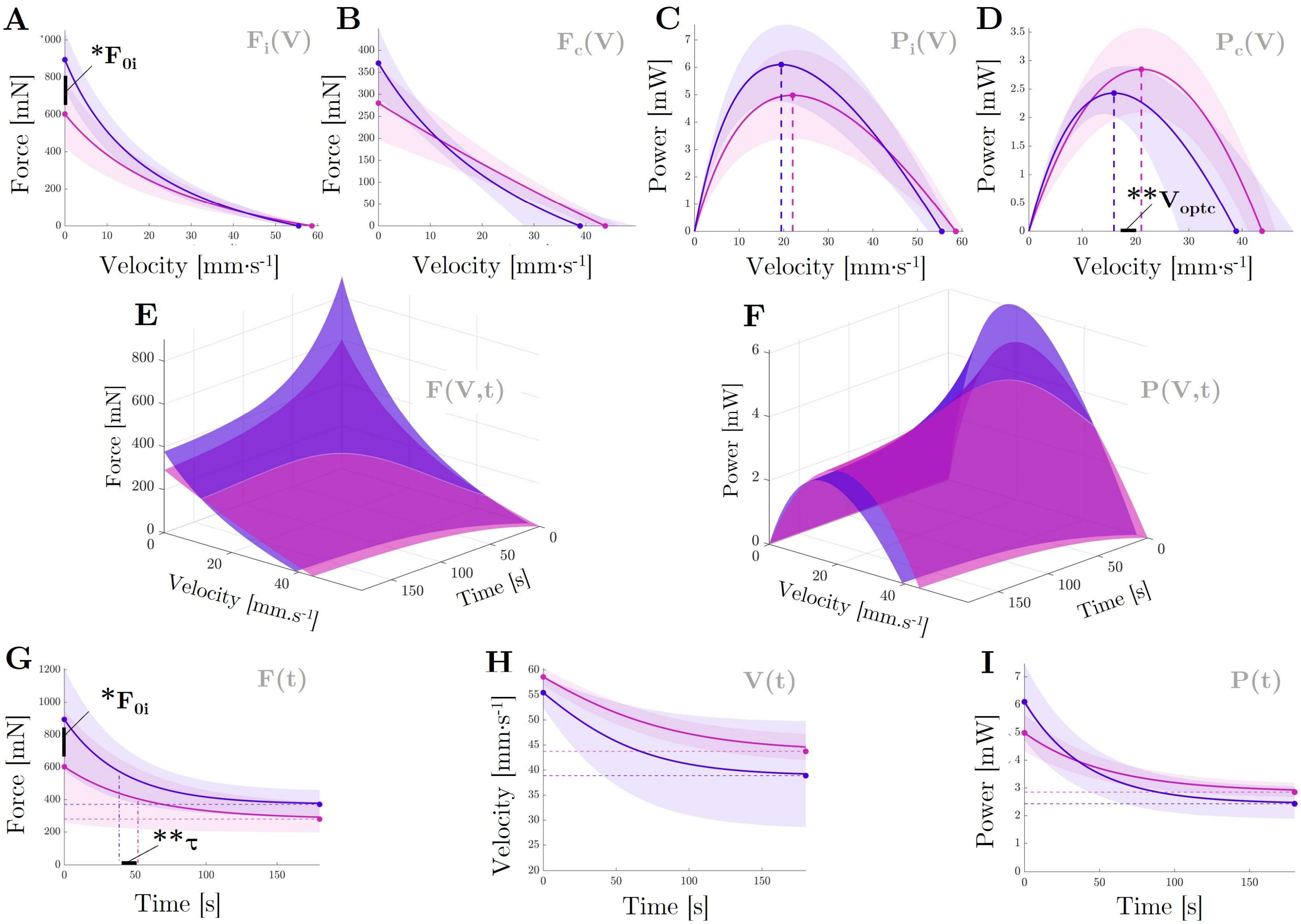
A
$F_i(V)$
*F_{0i}
Force [mN]
Velocity [mm·s-1]
B
$F_c(V)$
Force [mN]
Velocity [mm·s-1]
C
$P_i(V)$
Power [mW]
Velocity [mm·s-1]
D
$P_c(V)$
**V_{optc}
Power [mW]
Velocity [mm·s-1]
E
F(V,t)
Force [mN]
Velocity [mm.s-1]
Time [s]
F
P(V,t)
Power [mW]
Velocity [mm.s-1]
Time [s]
G
F(t)
*F_{0i}
**τ
Force [mN]
Time [s]
H
V(t)
Velocity [mm·s-1]
Time [s]
I
P(t)
Power [mW]
Time [s]

Supplementary Figure S7: Absolute 3D Force (E) and Power (F) -velocity-time relationships in ACTA1-MCM/+ (purple) and Acta1-MCM;FLExDUX4/+ mice (pink). 2D projections are presented : (A) initial force-velocity; (B) critical force-velocity; (C) initial power-velocity; (D) critical power-velocity; (G) maximal force-time; (H) maximal velocity-time; (I) maximal power-time. p values were calculated using GraphPad Prism9: 2-way ANOVA followed by a Fisher's LSD post hoc was performed. When significant, indices are represented graphically on 2D projections: F_{0i}: initial muscle maximal force in isometric condition (i.e. static, null velocity); V_{optc}: critical optimal contraction velocity (i.e. maximizing power production); τ: characteristic decay time (at τ seconds ≈63% of the final decay is reached); *$p < 0.05$; **$p<0.01$.
